# The Impact of Home Motor Affordances on Motor, Cognitive and Social Development of Young Children

**Published:** 2019

**Authors:** Asiye ZOGHI, Carl GABBARD, Masomeh SHOJAEI, Soheila SHAHSHAHANI

**Affiliations:** 1 Department of Physical Education, Shirvan Branch, Islamic Azad University, Shirvan, Iran; 2 Department of Health and Kinesiology, Texas A&M University, College Station, TX, USA; 3 Alzahra University, Department of Physical Education and Sport Sciences, Vanak St., Tehran, Iran; 4 Pediatric Neurorehabilitation Research Center, University of Social Welfare and Rehabilitation Sciences (USWR), Tehran, Iran.

**Keywords:** Affordances, Motor development, Cognitive development, Social development

## Abstract

**Objective:**

The present study evaluated the influence of home motor affordances on motor, cognitive, and social development of young children.

**Materials & Methods:**

The sample consisted of 49 Iranian children, aged 24-42 months. This study was conducted in Qouchan, Khorasan, Iran in 2015. They was randomly selected by multi-stage cluster sampling from a single community. Participant’s homes were assessed using the Affordances in the Home Environment for Motor Development (AHEMD). The motor behavior of young child was measured with the Denver Developmental Screening Test, aspects of cognitive development (Total cognitive, Verbal IQ, and non-verbal IQ) were assessed using the Stanford-Binet scale, and finally, social development measured by the Vineland Adaptive Behavior Scale.

**Results:**

Although no statistically significant correlations were found between Total AHEMD scores and motor development, there were significant and positive correlations (*P*=0.04) for Total AHEMD with total cognitive development (r=0.29), verbal IQ (r=0.29), social development (r=0.33) and (*P*=0.019), SES (r=0.51) with (*P*=0.000). There was a significant relationship between the Play Materials subscale of the AHEMD with total cognitive development (r=0.32) with (*P*=0.024), verbal IQ (r=0.31) and (*P*=0.029), and social development (r=0.35) with (*P*=0.012). In addition, there were significant differences between total AHEMD score with parents who had an academic education.

**Conclusion:**

Motor affordances in the home can have a significant positive influence on a young child’s cognitive and social development.

## Introduction

Motor development is a critical factor in child behavior, being associated with the foundation of cognitive and social-emotional development. In the developing infant, motor behavior is shaped by a combination of environmental, organismic, physiological, and genetic factors. Among those factors, the home environment has been established as a primary agent for learning and developing the foundation for positive lifelong behaviors (1). In general, rich environments have positive effects on brain development. There are critical periods in neuronal development in which experience may be the most effective in forging connections (wiring the brain). 

From another perspective, these critical periods more recently have been referred to as ‘windows of opportunity.’ That is the theory that nature opens certain windows for the experience effect starting before birth and then narrows each opportunity, one by one with increasing age. In theory, a series of windows exist for developing (for example) motor control, vision, language, and feelings. The child who misses an opportunity may not fully develop the brain’s circuitry to its optimal potential for a specific function. Contemporary research in child development suggests quite convincingly that an optimal level of development occurs in a stimulating environment and strong contextual support (2). Scientists now believe that to achieve the precision of the mature brain, neural function, and stimulation during infancy and early childhood are necessary. That is, optimal development is activity-dependent.

Perspective perceiving and experiencing are the same. The toddler as an active explorer perceives the environmental information and acts on it. The affordances of the environmental challenge the toddler's perception and action (2). Baffordances are opportunities for action that objects, events or places in the environment provide for the animal (3). The affordances include toys, materials, apparatus, availability of space, stimulation and nurturing would increase a toddler's development. Therefore, the home environment as an affordance can lead to optimal toddler development (4).

The home environment is a rich resource of opportunities that can be conducive to stimulating child development, especially at an early age. For most children, the interior of the home and its immediate surroundings are the first environments they experience throughout their early years. Children spend the majority of their time at home. Inside the home, kids have early interactions with family members and access to resources for learning and playing. Availability of stimulating objects, books, and toys in the home are critical indicators for the overall quality of the home environment (5). The home environment is certainly within the host of subsystems that contribute to infant motor development (6, 7). 

Supporting the home as a medium for growth and learning is ecological (affordance) theory. Although the term affordance has been interpreted in several ways, for this study it is one of a more general nature as suggested by Gibson (1979), "The affordances of the environment are what it offers the animal, what it provides or furnishes". In addition to the more clear set of affordances such as toys, materials, apparatus, and availability of space, stimulation and nurturing by parents (and others) produce the additional item of the events. Events are an affordance-events offer the child opportunities for action (8, 9). Using of the term affordance does not ignore the reciprocity between organism and environment, which frequently is addressed in experimental work. Since the intent was not to test the precise perceptual-motor mechanisms involved, reciprocity was not relevant (8, 9).

Since 2005, one of the most recognized efforts in determining the quality of the home for child motor development has been the *Affordances in the Home Environment for Motor Development – Self Report* (AHEMD-SR) for ages 18-42 months (4); a version is also available for infants 3-18 months, the AHEMD-IS (10). Both instruments have been used with several studies to examine the relationship between home motor affordances and child development. The validity of the AHEMD with an Iranian sample of children aged 18-42 months show that strong and significant multi-relationships between motor development and AHEMD scores. The best predictor of the AHEMD of overall motor ability was fine-motor toy availability (11). Another research study about the cultural adaptation and psychometric properties of the Persian version of the Affordance in the Home Environment for Motor Development (3-18 months (IS) and 18-42 month (SR) versions); both translated versions of the AHEMD were valid and reliable assessments of the home environment of Iranian young children (12-14). The majority of homes (80%-90%) provided good or very good affordances in respect to inside space, variety of stimulation, gross motor toys, fine-motor toys, and outside space (15). In a Japanese sample, both psycho-social and physical home affordances influenced young children’s motor development (16).

The relationship examined between home motor affordances, motor development, and cognitive ability. Results showed a positive correlation between cognitive performance and fine motor performance and found that motor affordances had a positive influence on future motor ability and cognitive behavior (17). Whereas home motor affordances were not examined, other studies indicated a clear relationship between the home environment and cognitive outcomes of young children (18-22).

Due to lack of research, especially in the field of social development, we decided to examine the association between home motor affordances and motor, cognitive, and social development of young children aged 24-42 months. We also included socioeconomic status (SES) characteristics of the family. Our interest in these questions was derived in part from the work which examined the relationship between motor affordances in the home and cognitive development of infants. As new research, we examined the effects of home motor affordances on social development. The significance of this study relates to the need to identify factors that may contribute or constrain motor, cognitive, and social ability in young children. Such information could be helpful in providing opportunities for developmental change earlier in life, especially for young children that are at risk for developmental delays. Moreover, the home and its varied dimensions had a significant relationship with the status of fine- and gross motor skill behavior. SES (income, parental education) was associated with the level of affordances in the home (14).

## Materials & Methods

The sample (49 young children, age range: 24-42 months) selected randomly from the statistical population, using a multistage cluster sampling method. The potential sampling pool consisted of healthy children with average family’s SES (using Kuppuswamy's SES scale) located in Iran. The sample size was determined using the G-power statistical software. 

This research was approved by the Ethics Medical Board of Islamic Azad University of Shirvan.

For assessment of the home affordances, we used the* Affordances in the Home Environment for Motor Development-Self Report (AHEMD)* (4); a reliable and valid parental self-report assessment instrument that addresses the home opportunities for infants and young children (ages 18-42 months). The instrument consists of five factors (subscales): Inside Space, Outside Space, Variety of Stimulation, Fine Motor Toys, and Gross Motor Toys, plus a section on Child and Family Characteristics. For simplicity of presentation, we combined Outside Space and Inside Space into one dimension of Physical Space as well as a combination for Fine-Motor and Gross-Motor Toys, which resulted in the larger dimension of Play Materials. Scoring for each dimension was calculated by summing up all points obtained for each question within each dimension (Physical Space [0–16], Variety of stimulation [0–25], and Play Materials [0–126]. The total score is the sum of scores of the three dimensions [0–167]. In Iran, the *AHEMD *content-related validity was 0.92. Reliability over time was 0.91 and internal consistency was 0.93. Fne- and gross-motor toys showed a signiﬁcant 55% predictability of affordance provision in the home (14). 

The instrument used for the assessment of motor development was the *Denver Developmental Screening Test *(DDST-II). The DDST-II is one of the most extensively used screening instruments for children from birth to 6 years of age. The test was standardized on 2096 children with a test-retest reliability of 90% and inter-rater reliability of 80%-95%. The DDST-II assesses children’s development in four general areas: Personal–social (25 items), Fine motor-adaptive (29 items), Language (39 items), and Gross motor (32 items). We used the Persian version of the DDST-II with accepted validity and reliability (23).

For the measurement of cognitive development, we used the *Stanford-Binet Intelligence Scales: Fifth Edition *(SB-5) which is a test of intelligence/cognitive abilities for individuals ages 2 - 85 yr. The SB-5 was a major revision and purports to measure 10 subtests with five factors, within Verbal (five subtests) and Nonverbal (five subtests) domains. Validity for total and subscales range from 0.84 to 0.98. Inter-rater and test-retest reliability coefficients are higher than 0.75 (24).

The *Vineland Social Development Scale* was used to measure social development. This scale has an age range of infancy to 25 yr and is divided into eight subscales (general self-help (SA), eating self-help (SQ), dressing self-help, self-direction, occupation, communication, locomotion, and sociability). This scale estimates the social age and social intelligent quotient (25).

In regard to procedure, first, out of eight health centers in the selected town, five were selected randomly for this research. Visits were made, and the goals of the research were described to the directors. With the facility director’s approval and cooperation, contact with qualified potential research participants (families) was made. That is, families whose children met the age, health, and cognitive criteria. Contact included a written description of the study and phone communication. Consent forms and the SES questionnaire were distributed to the parents during the visit. The AHEMD questionnaire, Stanford-Binet scale, Denver Developmental Screening Test and Vineland Adaptive Behavior Scale were completed by a trained researcher with the help of the parents in their homes.

 Pearson’s correlation tests were used for calculation of the associations between total AHEMD and subtest scores with cognitive and social behavior (total and subscales). Analysis of variance procedure was used for evaluation of the relation between children’s average verbal intelligence and education levels of parents. All statistical tests were performed at a significance level of α=.05 using the SPSS software version 15 (Chicago, IL, USA).

## Results

Overall, 49 young children (51% were female) (homes) participated in the study. The majority of participants were ‘an only child’ (41%), 45% lived with one sibling, and 14% had more than one sibling. In addition, 65% were not attending day-care and 35% were enrolled. Fifty-five percent lived in apartments and 94% lived in free-standing homes. Seventy-four percent of families had low SES, while 16% were medium, and 10% high. Regarding education, 10% of mothers completed elementary school, 37% high school, and 53% had academic degrees. In reference to fathers, 14% completed elementary school, 37% completed high school, and 49% had academic degrees.

There was a positive relationship between the total AHEMD score and cognitive development, more specifically, verbal IQ, *r*=0.295. Furthermore, there was a positive and significant correlation between total AHEMD score and social development (*r*=0.335) (Table 1).

**Table 1:** The relationships between total AHEMD score, cognitive and social development

Type of Developments	AHEMD score	*P*-value
Verbal IQ	0.295	0.04
Cognitive development	0.295	0.04
Social development	0.335	0.019

* Correlation is significant at the 0.05 level

Table 2 shows the correlation values for the Play Material subscale and selected factors. There were positive significant associations with verbal IQ (*r*=0. 31), cognitive development (*r*=0.32) and social development (*r*=0.35).

**Table 2:** The relationships between Play Material subscale and cognitive and social development

Type of Developments	Play Material	*P*-value
Verbal IQ	0.312	0.029
Cognitive development	0.322	0.024
Social development	0.357	0.012

* Correlation is significant at the 0.05 level

There was a significant difference between the AHEMD score of children with that their parents had a higher educational level (academic education and children of a parent with versus primary school education). ANOVA results for AHEMD score and mothers and fathers' education levels indicated a significant difference, F (2, 46)=4.40, *P*<0.05 and F (2, 46)=7.96, *P*<0.001, respectively.

Table 3 shows correlation values between SES, total AHEMD, and subscales. All associated were significant and in a positive direction. The value for total AHEMD and SES was (0.51), Physical Space and AHEMD (0.35), and Play Material and SES (0.47).

**Table 3:** Pearson correlation between SES, total AHEMD and subscales

Variables	Pearson correlation	*P*-value
Total AHEMD-SES's score	0.512**	0.000
Physical space-SES's score	0.358*	0.012
Play material-SES's score	0.472**	0.001

* Correlation is significant at the 0.05 level

** Correlation is significant at the 0.01 level

These results indicate a significant correlation between SES of the family and motor affordances in the home.

## Discussion

The present study investigated the effect of home motor affordances on the motor, cognitive, and social development of children ages 24-42 months. In our study, there was a significant correlation between total AHEMD score with cognitive development and verbal IQ. In addition, there was a meaningful correlation between the Play Material subscale with cognitive development and verbal IQ. This information supports other studies showing a strong relationship between the home environment with cognitive ability (7, 17-19, 21, 22, 26, 27).

Of the findings noted in this study, the most prominent is the positive effect of home motor affordances on the child's social development. This result is the most interesting and unique contribution to the study. Although motor affordances were not included in past studies, there is a hint of support that a strong positive relationship is between the home environment with cognitive and social development ability (5, 20, 28-30). For instance, a Korean study reported that children’s self-perceived competence and home environment stimulation were positively correlated (28).

Obviously, we did not find a significant positive association between the home motor affordances and motor development of our sample. A few possible explanations come to mind. Foremost is the likelihood that the Denver II test was not sensitive enough to detect differences – that is, in our case the test was too easy for the sample. Our finding for that aspect of the study is in opposition to several reports (11, 16, 17, 31-33). In comparison to our results, in Iran, total AHEMD score was the best predictor for motor development (11). Moreover, in Japan, total AHEMD scores were positively associated with young children’s motor development (16).

Another finding was significant and positive correlations values between SES with total AHEMD, Physical Space, and Play Material. In line with our results they evaluated the availability of affordances in the home to promote infant motor development and family SES. Results indicated a significant influence of SES indicators on the availability of physical space and play materials. Furthermore, the physical space of the home was influenced by family SES. The Play Materials dimension influenced by all SES indicators (34).

There were significant differences between the AHEMD scores of children with parents who had higher academic education and children of a parent with primary education. Parental education, especially maternal education, could play a significant role in child development (34-36). For example, play materials and total AHEMD score were influenced by the parents’ education level (34). For many families, income and education were major environmental constraints that could positively impact aspects of the home environment, including the availability of motor affordances.

**In Conclusion, **home motor affordances have a positive influence on the cognitive and social development of young children. Parents and caretakers provide and take advantage of home affordances to promote their child’s development. In regard to study limitations and future work, we suggest using larger sample size and a more stringent assessment of motor development. The Denver II may not be sensitive enough to detect fine- and gross motor as it is associated with home affordances. Exploring the quality of the home environment and its impact on infant development may provide a fundamental clue to understanding the complex nature of human development.

## References

[1] Caçola PM, Gabbard C, Montebelo MI, Santos DC (2015). The new affordances in the home environment for motor development-infant scale (AHEMD-IS): Versions in English and Portuguese languages. Braz J Phys Ther.

[2] Gabbard, Caçola, Spesatto, Santos (2012). The Home Environment And Infant And Young Children’s Motor Development. Adv Psychol Res.

[3] Gabbard, Caçola, Rodrigues (2008). A new inventory for assessing affordances in the home Environment for Motor Development (AHEMD-SR). Early Childhood Educ J.

[4] Rodrigues, Saraiva, Gabbard (2005). Development and construct validation of an inventory for assessing the home environment for motor development. Res Q Exerc Sport.

[5] Iltus S (2007). Significance of home environments as proxy indicators for early childhood care and education.

[6] Abbott AL, Bartlett DJ, Fanning JEK, Kramer J (2000). Infant Motor Development and Aspects of the Home Environment. Pediatr Physic Ther.

[7] Bradley RH, Caldwell BM, Rock SL (1989). HOME environment and cognitive development in the first 3 years of life: a collaborative study involving six sites and three ethnic groups in North America. Develop Psychol.

[8] Stoffregen TA (2000). Affordances and events. Ecological psychology.

[9] Hirose N (2002). An ecological approach to embodiment and cognition. Cognitive Systems Research.

[10] Caçola PM, Gabbard C, Montebelo MI, Santos DC (2015). Further development and validation of the affordances in the home environment for motor development–infant scale (AHEMD-IS). Phys Ther.

[11] Haydari A, Askari P, Nezhad MZ (2009). Relationship between affordances in the home environment and motor development in children age 18-42 months. J Soc Sci.

[12] Kavousipor S, Rassafiani M, Ebadi A, Solimani F, Hosseini A, Gabbard C (2019). Cultural adaptation and psychometric properties of the Persian version of the Affordance in the Home Environment for Motor Development. Iran J Child Neurol.

[13] Kavousipor S, Nejad SF, Jamali E, Ghahfarokhi SZ (2017). Test-retest Reliability of the Persian version of the questionnaire Affordances in the Home Environment for Motor Development-Infant Scale and its Relation with Infant Movement. J Rehabil Sci Res.

[14] Valadi S, Gabbard C, Arabameri E, Kashi A, Ghasemi A (2018). Psychometric properties of the Affordances in the Home Environment for Motor Development inventory for use with Iranian children aged 18–42 months. Infant Behav Dev.

[15] Temple VA, Naylor P-J, Rhodes RE, Higgins JW (2009). Physical activity of children in family child care. Appl Physiol Nutr Metabol.

[16] Mori S, Nakamoto H, Mizuochi H, Ikudome S, Gabbard C (2013). Influence of Affordances in the Home Environment on Motor Development of Young Children in Japan. Child Develop Res.

[17] Miquelote, Santos, Caçola, Montebelo, Gabbard (2012). Effect of the home environment on motor and cognitive behavior of infants. Infant Behav Dev.

[18] Biedinger N (2011). The influence of education and home environment on the cognitive outcomes of preschool children in Germany. Child Develop Res.

[19] Espy KA, Molfese VJ, DiLalla LF (2001). Effects of environmental measures on intelligence in young children: Growth curve modeling of longitudinal data. Merrill-Palmer Quarterly (1982-).

[20] Parks PL, Bradley RH (1991). The interaction of home environment features and their relation to infant competence. Infant Mental Health Journal.

[21] Zoghi A, Shojaei M, Ghasemi A (2016). The Impact of a Motor Affordance Intervention on Motor and Cognitive Development of Young Children. Int J Mental Health Addic.

[22] Tong S, Baghurst P, Vimpani G, McMichael A (2007). Socioeconomic position, maternal IQ, home environment, and cognitive development. J Pediatr.

[23] Shahshahani S, Sajedi F, Azari N, Vameghi R, Kazemnejad A, Tonekaboni S-H (2011). Evaluating the Validity and Reliability of PDQ-II and Comparison with DDST-II for Two Step Developmental Screening. Iran J Pediatr.

[24] Roid GH, Barram RA ( 2004). Essentials of Stanford-Binet intelligence scales (SB5) assessment.

[25] Pedrini D, Pedrini BC (1973). An Evaluation of and a Detail-Profile for the Vineland Social Maturity Scale.

[26] Ramey CT, Ramey SL (1994). Which children benefit the most from early intervention?. Pediatrics.

[27] Shojaeian N-A, Shojaei M, Ghasemi A (2017). Exercising during pregnancy: An experimental study of its effects on cognitive development in early infancy. Women.

[28] Lee J, Super CM, Harkness S (2003). Self‐perception of competence in Korean children: Age, sex and home influences. Asian J Soc Psychol.

[29] Werner E, Simonian K, Bierman JM, French FE (1967). Cumulative effect of perinatal complications and deprived environment on physical, intellectual, and social development of preschool children. Pediatrics.

[30] Zoghi A, Shojaei M, Ghasemi A (2016). The Effect of an Environment Motor Affordance Intervention on Social Development of Toddlers. J Develop Motor Learning.

[31] Hsieh Y-h, Hwang A-w, Liao H-f, Chen P-c, Hsieh W-s, Chu P-y (2011). Psychometric properties of a Chinese version of the home environment measure for motor development. Disability and Rehabilitation.

[32] Valadi S Comparison of Affordances in the Home Environment for Motor Development with the Economic Situation and Parent Level of Education.

[33] Valadi S, Gabbard C (2018). The effect of affordances in the home environment on children’s fine-and gross motor skills. Early Child Development and Care.

[34] Freitas TC, Gabbard C, Cacola P, Montebelo MI, Santos DC (2013). Family socioeconomic status and the provision of motor affordances in the home. Braz J Phys Ther.

[35] Bradley RH, Corwyn RF (2002). Socioeconomic status and child development. Ann Rev Psychol.

[36] Lung F-W, Chiang T-L, Lin S-J, Feng J-Y, Chen P-F, Shu B-C (2011). Gender differences of children's developmental trajectory from 6 to 60 months in the Taiwan Birth Cohort Pilot Study. Res Dev Disabil.

